# Mesoscale Damage Evolution, Localization, and Failure in Solid Propellants Under Strain Rate and Temperature Effects

**DOI:** 10.3390/polym17152093

**Published:** 2025-07-30

**Authors:** Bo Gao, Youcai Xiao, Wanqian Yu, Kepeng Qu, Yi Sun

**Affiliations:** 1 College of Mechatronic Engineering, North University of China, Taiyuan 030051, China; zbdx2773@163.com; 2 National Key Laboratory of Energetic Materials, Xi’an Modern Chemistry Research Institute, Xi’an 710065, China; qukepeng204@163.com; 3 National Key Laboratory of Land and Air Based Information Perception and Control, Xi’an Modern Control Technology Research Institute, Xi’an 710065, China; hsywq812907@163.com; 4 Departments of Astronautic Science and Mechanics, Harbin Institute of Technology, Harbin 150001, China; sunyi@hit.edu.cn

**Keywords:** high-energy solid propellants, cohesive finite element model, neural network, temperature effect, crack extension mode

## Abstract

High-energy solid propellants are multiphase engineering materials, whose mechanical behavior is predominantly governed by the characteristics of embedded crystalline particles. While microstructural influences have been extensively examined, quantitative correlations between microstructure and macroscopic mechanical properties remain underexplored. This work develops a cohesive finite element method (CFEM) framework to quantify the thermomechanical response of high-energy solid propellants at the microstructural scale. The analysis focuses on impact loading at strain rates ranging from 10^3^ to 10^4^ s^−1^, accounting for large deformation, thermomechanical coupling, and microcrack-induced failure. Damage evolution under impact conditions was evaluated using a combined neural network-based inverse identification method and a three-dimensional cohesive finite element model to determine temperature-dependent bilinear-polynomial cohesive parameters. Results demonstrate a strong dependence of the propellant’s mechanical behavior on both strain rate and temperature. Validation against experimental data confirms that the proposed temperature-sensitive CFEM accurately predicts both damage progression and macroscopic mechanical responses.

## 1. Introduction

Solid propellants, such as hydroxyl-terminated polybutadiene (HTPB) and nitrate ester plasticized polyether (NEPE), exemplify highly filled particulate composites. These materials primarily consist of rigid energetic constituents—including ammonium perchlorate (AP), hexogen (RDX), and hexanitrohexaazaisowurtzitane (CL-20)—dispersed in a lightly cross-linked viscoelastic binder [1,2,3]. As the core material in solid rocket motors, the mechanical performance of these propellants is critical to maintaining structural stability under operational loads [4,5,6]. Accurate characterization of their mechanical response under diverse loading conditions remains a key research objective. Despite substantial efforts to develop constitutive models, significant challenges persist due to nonlinearities introduced by evolving damage mechanisms [7,8,9]. Variations in temperature affect the binder’s properties, thereby altering the kinetics and morphology of damage accumulation. This temperature dependence influences both the initiation and evolution of fine-scale damage, impacting the material’s time-dependent mechanical behavior [10,11,12]. Therefore, investigating the temperature-dependent mechanical response of solid propellants is essential for advancing predictive modeling.

Computational methods—particularly finite element and meshfree approaches—have been widely adopted to simulate failure processes in solid propellants. Cohesive zone models (CZMs) have been applied to capture interfacial failure phenomena [13,14,15]. During loading, substantial mismatches in stiffness between particles and the binder matrix result in stress concentrations at interfaces, leading to interfacial debonding and microcrack formation [16,17,18,19]. Increased particle packing density tends to elevate porosity levels introduced during fabrication. Under mechanical loading, these pores often coalesce with microcracks, promoting damage propagation and culminating in macroscopic crack formation, which significantly degrades mechanical performance. While earlier studies primarily emphasized qualitative or phenomenological observations, a more comprehensive understanding necessitates quantitative analysis of damage progression. To this end, fracture mechanics principles have been integrated into numerical frameworks to examine the mechanical and damage characteristics of propellants and particle-reinforced composites [20,21,22]. Recent advances in mesoscale damage modeling have facilitated a deeper exploration of damage mechanisms and their macroscopic implications. These efforts incorporate both surface-level characterization techniques—such as optical microscopy (OM) [23] and scanning electron microscopy (SEM) [24]—and internal damage tracking via high-resolution X-ray computed tomography (CT). Hu et al. [25] analyzed the effects of micropore expansion on bulk mechanical properties, establishing links between microstructural features and macroscopic behavior. Subsequent studies employed CZMs for quantitative simulation of damage evolution, further bridging the gap between microscale phenomena and macroscale mechanical degradation in propellants [15,26,27]. However, existing studies still have two key limitations. First, most cohesive zone models (CZMs) remain primarily two-dimensional [28,29,30]. Such models are only applicable to preliminary analyses involving geometrically simple structures, planarized loads, and low precision requirements. Second, the majority of existing methods for obtaining cohesive zone element parameters rely on the Kalman filtering algorithm [31], which depends on accurate system models and noise assumptions. This renders it constrained in scenarios characterized by complexity, nonlinearity, or unknown models.

To address the aforementioned issues, this study investigates the compressive damage behavior of solid propellants under high strain rates and varying temperature conditions using a framework based on a three-dimensional thermo-mechanical cohesive zone model. An artificial neural network (ANN) model is employed to determine the parameters of the continuum finite element model (CFEM). The performance of the neural network system and the effectiveness of the proposed model are evaluated by comparison with experimental results. Damage mechanisms across a range of strain rates are examined and quantitatively characterized. Compared with existing works, the method presented in this paper enables more accurate prediction of the damage evolution process of propellants under different temperature and strain rate conditions.

## 2. Model and Methodology

The constitutive model of each component was defined. A linear elastic model was applied to crystalline phases, while the binder was modeled as a linear viscoelastic material. To simulate damage in the crystals, binder and interfaces, zero-thickness cohesive elements governed by a bilinear traction–separation law were incorporated throughout the structure. Parameters for the bilinear model were determined using a hybrid inverse optimization strategy.

### 2.1. Viscoelastic Model for Polymer Binder

The generalized Maxwell model (GMM) was used to represent the linear viscoelastic behavior of the polymer binder. The model, expressed in integral form, was derived from the Boltzmann superposition principle, as shown in Equation (1) [32]:

(1) $$\sigma_{ij}(t)=L_{ijkl}^{e}\varepsilon_{kl}+\int_{0}^{t}L_{ijkl}(t-\tau )\frac{\partial \varepsilon_{kl}(\tau )}{\partial \tau }d\tau$$

where $\sigma_{ij}(t)$ and $\varepsilon_{kl}(t)$ are the instantaneous stress and strain tensors, respectively, $L_{ijkl}^{e}$ is the equilibrium relaxation modulus tensor, and $L_{ijkl}$ is the relaxation modulus tensor. $L_{ijkl}$ is expressed using Equation (2):

(2) $$L_{ijkl}=[K(t)-\frac{2}{3}\mu (t)]\delta_{ij}\delta_{kl}+\mu (t)[\delta_{ik}\delta_{jl}+\delta_{il}\delta_{jk}]$$

where K(t) and μ(t) are the bulk and shear moduli, respectively.

The relaxation functions were modeled using a Prony series expansion. Both the shear modulus *G*(*t*) and the bulk modulus *K*(*t*) were expressed as follows:

(3) $$\left\{ \begin{array}{l} G\left(t\right)=G^{\left(\infty \right)}+\sum_{n=1}^{N}G^{\left(n\right)}\exp\left(-\frac{t}{\tau^{\left(n\right)}}\right)\\ K\left(t\right)=K^{\left(\infty \right)}+\sum_{n=1}^{N}K^{\left(n\right)}\exp\left(-\frac{t}{\tau^{\left(n\right)}}\right) \end{array}\right.$$

where *G*^(*n*)^ and *K*^(*n*)^ represent the shear and bulk stiffnesses, respectively, $\tau^{\left(n\right)}$ represents the shear and bulk relaxation times, *G*^(∞)^ and *K*^(∞)^ are the long-term elastic shear and bulk moduli, respectively, and *N* is the number of Maxwell elements.

### 2.2. Thermomechanical Cohesive Zone Element

**Figure 1:**
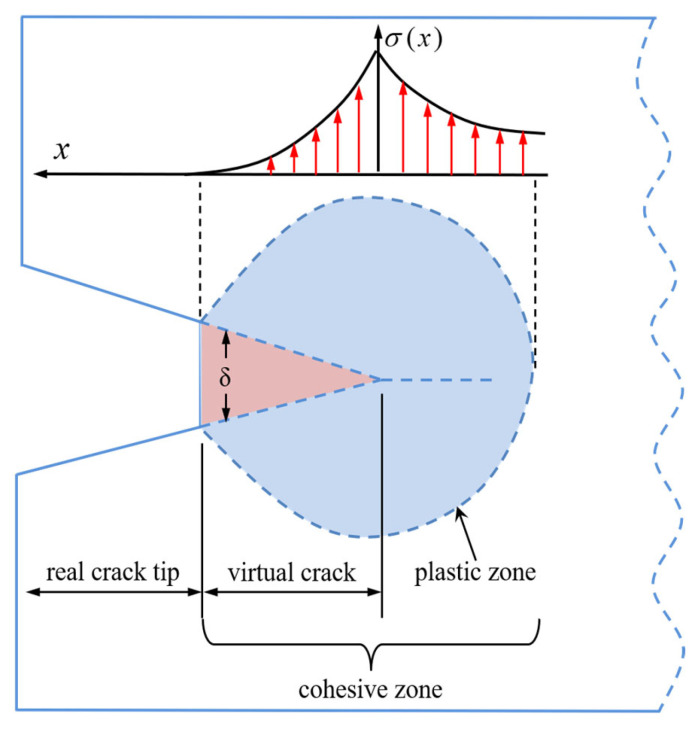
Schematic of the crack tip region under the cohesive zone model.

The method proposed by Xu and Needleman [33] was employed to insert cohesive elements between physical elements to simulate crack propagation. As illustrated in Figure 1, the cohesive zone comprises virtual cracks and a plastic zone, with real cracks forming beyond the cohesive zone. No stress was assumed on surfaces adjacent to real cracks, and the stress field *σ*(*x*) at any point within the plastic zone depended on the distance from the crack tip. Stress evolution followed a traction–separation law, expressed as a function of the cracking displacement *S*(*δ*). When the stress exceeded a critical threshold, the stiffness of the virtual crack diminished, eventually forming a real crack.

To analyze damage and failure mechanisms under thermal expansion at fine scales, the thermomechanical response of cohesive elements was investigated. This analysis incorporated thermal–mechanical coupling effects to capture the initiation and evolution of microscopic defects. The failure mechanism progressed through three stages: thermally induced stress, damage initiation, and crack propagation. Thermal mismatch stresses arose at interfaces due to differences in the coefficients of thermal expansion among material phases. The thermal strain for each component was calculated using the following:

(4) $$\varepsilon_{\mathrm{thermal}}^{\left(i\right)}=\alpha^{\left(i\right)}\Delta T(i=1,2,\ldots )$$

where α is the thermal expansion coefficient, and ΔT are the temperature changes.

The interfacial failure strain was defined as follows:

(5) $$\Delta \varepsilon_{interface}=\varepsilon_{\mathrm{thermal}}^{\left(1\right)}-\varepsilon_{\mathrm{thermal}}^{\left(2\right)}$$

where $\varepsilon_{\mathrm{thermal}}^{\left(1\right)}$ and $\varepsilon_{\mathrm{thermal}}^{\left(2\right)}$ represent the thermal expansion strains of the matrix and grain phases, respectively. Among them, the grain phase refers to the energetic particles dispersed in the propellant, and the matrix phase refers to the polymer binder in the propellant.

Normal and shear stresses in the cohesive element increased linearly with interfacial separation. The slopes of these relationships defined the stiffness of the cohesive element. Upon reaching the critical normal and shear stress values, *S_n_*_max_ and *S_t_*_max_, damage evolution was initiated according to

(6) $${\left(\frac{S_{n}}{S_{n\max}(T)}\right)}^{2}+{\left(\frac{S_{t}}{S_{t\max}(T)}\right)}^{2}\geq 1$$

where $S_{n}$ and $S_{t}$ are the temperature-dependent normal and shear stresses, respectively.

Cohesive element separation was controlled using the damage variable, defined as follows:

(7) $$S_{n}=\left\{\begin{array}{l} (1-D)\chi_{t}\delta_{t}\\ (1-D)\chi_{n}\delta_{n} \end{array}\right.$$

where *D* is damage variable, $\delta_{n}$ and $\delta_{t}$ are the normal and shear displacement of the cohesive element, and $\chi_{t}$ and $\chi_{n}$ are the normal and shear temperature correction parameter of the cohesive element.

### 2.3. Fine-Grained Modeling

**Figure 2:**
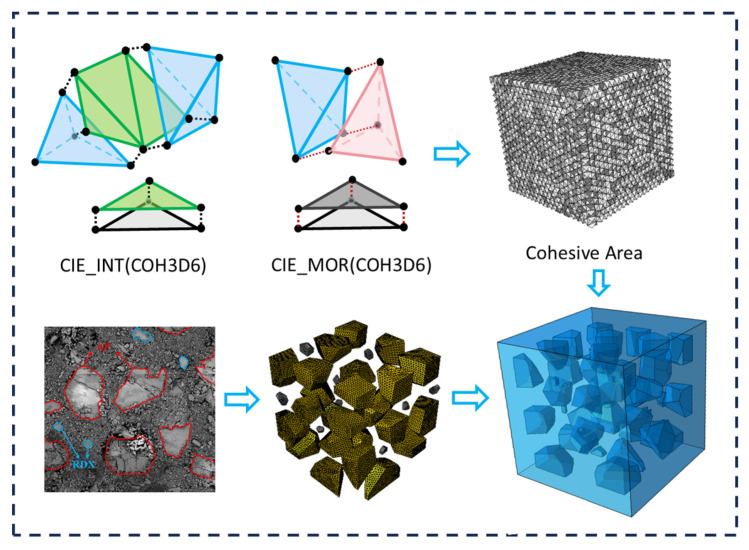
3D fine-scale model of the composite propellant.

The investigated HTPB propellant is a particle-reinforced composite comprising 41 wt.% AP, 9.2 wt.% cyclotrimethylene trinitramine (RDX), 48 wt.% HTPB matrix, and additional minor additives. The typical morphology of AP and RDX crystals observed under scanning electron microscopy (SEM) is shown in Figure 2. Most AP particles exhibit a near-spherical shape with diameters ranging from 200 to 300 μm; a minority are smaller than 100 μm and display relatively smooth contours. By contrast, RDX particles are generally finer, with diameters between 40 and 60 μm. Previous studies on the microscale mechanical behavior of solid propellants have predominantly utilized two-dimensional cohesive zone models. However, to more accurately capture the effects of particle morphology and spatial distribution on mechanical responses—particularly in relation to crack initiation, propagation, and damage mechanisms—a three-dimensional (1 × 1 × 1 mm) fine-scale representative volume element was constructed. After conducting a grid convergence analysis on the numerical simulation model, the optimal grid size was determined to be 0.012 mm. At this mesh size, the computer calculation time is 18.2 h, and the peak stress of the model is basically converged to a fixed value. When the mesh size increases, the peak stress of the model is lower; when the mesh size decreases, the calculation time is longer. Therefore, the optimal mesh size for the numerical simulation model in this study is determined to be 0.012 mm. Based on the particularity of the particle shape in this three-dimensional model, we selected the 6-node three-dimensional cohesive element. Compared with the 4-node and 8-node cohesive elements, the 6-node cohesive element can significantly reduce the calculation time, enhance the bending degree of freedom, and accurately predict the interface failure. Zero-thickness cohesive elements were embedded at the interfaces and interiors of AP, RDX, and the HTPB matrix to simulate interfacial interactions and fracture evolution.

## 3. Model Validation

### 3.1. Modeling Parameters

**Figure 3:**
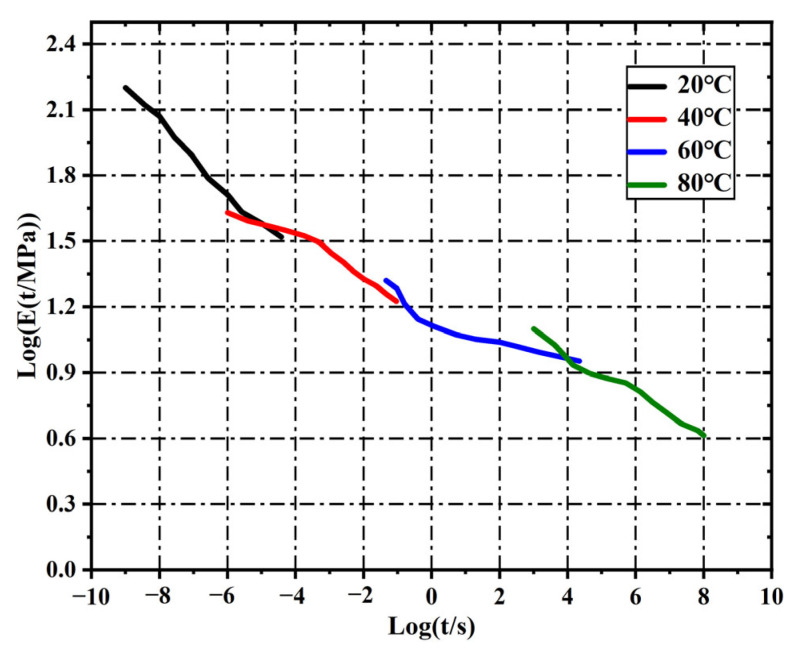
Master curve of relaxation modulus for HTPB at variable temperatures. Here, *E*(*t*) is relaxation Young’s modulus.

The oxidizer crystals in solid propellants, namely ammonium perchlorate (AP) and RDX, are generally classified as quasi-brittle materials, exhibiting negligible plastic deformation upon fracture. Both AP and RDX particles were modeled as linear elastic solids. The Young’s modulus and Poisson’s ratio for AP were set at 32.4 GPa and 0.13, respectively [34] while, for RDX, the corresponding values were 8.6 GPa and 0.3 [35]. HTPB, a polymeric binder, displays significant viscoelastic behavior, including pronounced creep and stress relaxation, as demonstrated in extensive prior studies [32,36]. Based on experimental data from previous relaxation tests on HTPB [37], a master curve of the relaxation modulus was constructed using the time–temperature superposition principle, as shown in Figure 3. The master curve was fitted using an 18th-order Prony series. The fitted parameters are listed in Table 1. In the Prony model, *E*(*t*) denotes the time-dependent Young’s modulus. The corresponding bulk modulus *K_i_* and shear modulus *G*_i_ were derived from the elastic modulus components using the relations $K_{i}=\frac{E_{i}}{3(1-2\nu )}$ and $G_{i}=\frac{E_{i}}{2(1+\nu )}$, where the Poisson’s ratio (ν) is 0.45.

### 3.2. Mechanical Characterization of Solid Propellants

**Figure 4:**
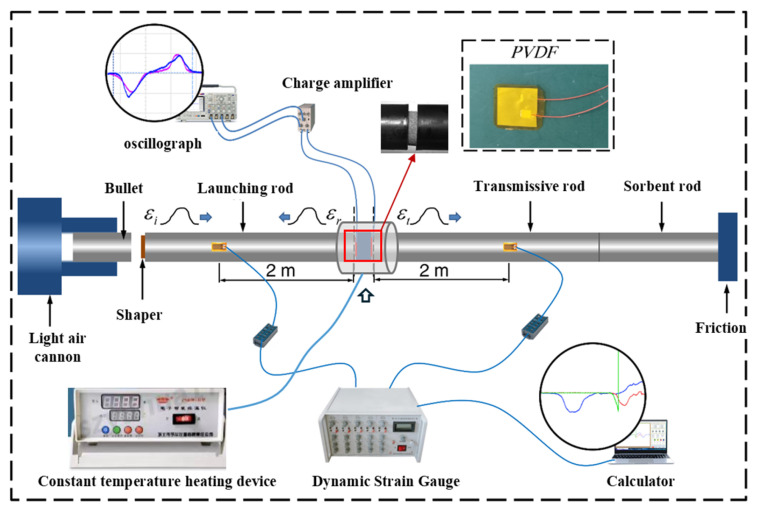
Schematic of the SHPB system with temperature control.

Dynamic compressive properties were evaluated using a split Hopkinson pressure bar (SHPB), a standard apparatus for high strain rate testing of energetic materials [38,39,40]. The SHPB system included an impact bar, incident bar, transmission bar, and absorption bar, as illustrated in Figure 4. Due to the low density and acoustic velocity of the propellant, aluminum was selected for the bars to reduce impedance mismatch. The aluminum bars had a Young’s modulus of 73 GPa and a density of 2700 kg/m^3^. The lengths of the impact, incident, transmission, and absorption bars were 300 mm, 3000 mm, 3000 mm, and 1500 mm, respectively, each with a diameter of 20 mm.

**Figure 5:**
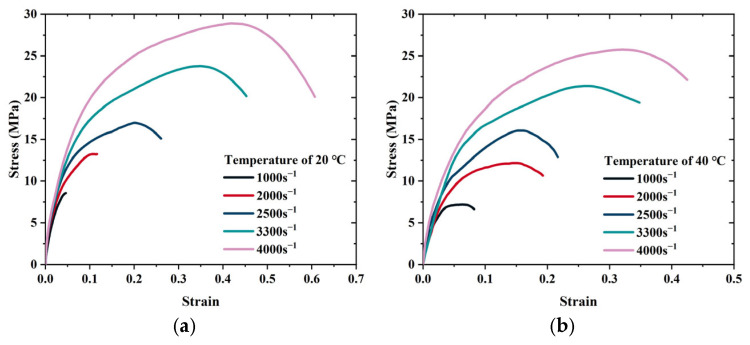

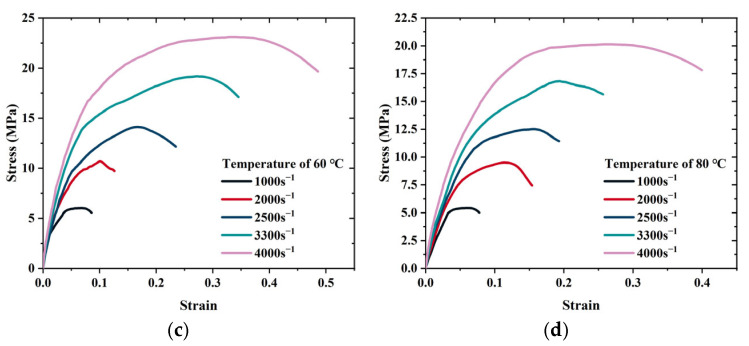
Stress–strain responses of the propellant under varying temperatures and strain rates. (**a**) 20 °C, (**b**) 40 °C, (**c**) 60 °C and (**d**) 80 °C.

Figure 5 presents the stress–strain curves of the solid propellant at various strain rates and temperatures, demonstrating typical viscoelastic behavior. Because of the high binder content, the material exhibited significant nonlinearity and toughness during deformation. At 20 °C, increasing the strain rate from 1000 s^−1^ to 4000 s^−1^ elevated the peak yield stress from 8.23 MPa to 28.64 MPa, a 3.48-fold increase. At a constant strain rate of 2000 s^−1^, raising the temperature from 20 °C to 80 °C reduced the elastic modulus from 131.82 MPa to 93.65 MPa, indicating a maximum reduction of 14.2%, consistent with temperature-dependent softening behavior.

**Figure 6:**
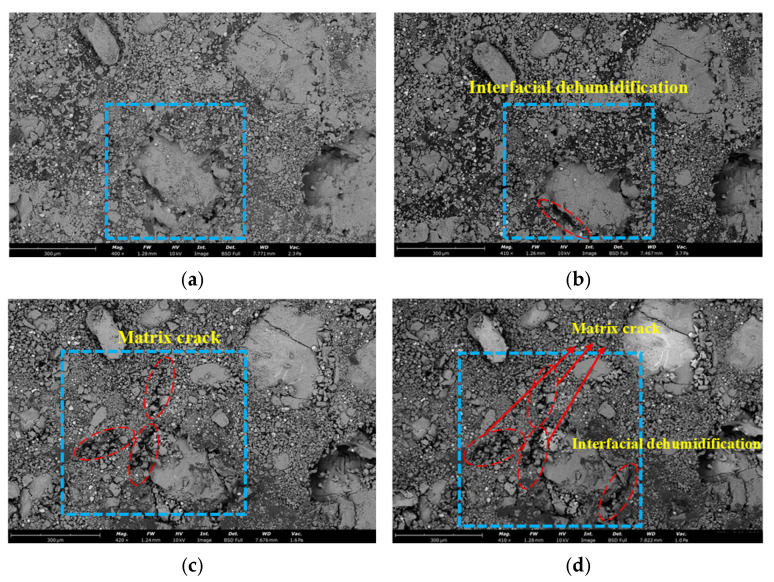
SEM images of the propellant microstructure at different temperatures. (**a**) 20 °C, (**b**) 40 °C, (**c**) 60 °C and (**d**) 80 °C.

As shown in Figure 6, SEM imaging revealed microstructural changes in the propellant from 20 °C to 80 °C. Interfacial dehumidification became increasingly prominent with temperature. Initial damage localized at the particle–binder interface. With increasing temperature, cracks initiated at these interfaces propagated into the binder matrix, forming microcracks. Thermal expansion of the binder contributed to volumetric increase and growth of internal voids.

### 3.3. Inverse Optimization for Cohesive Model Parameters

**Figure 7:**
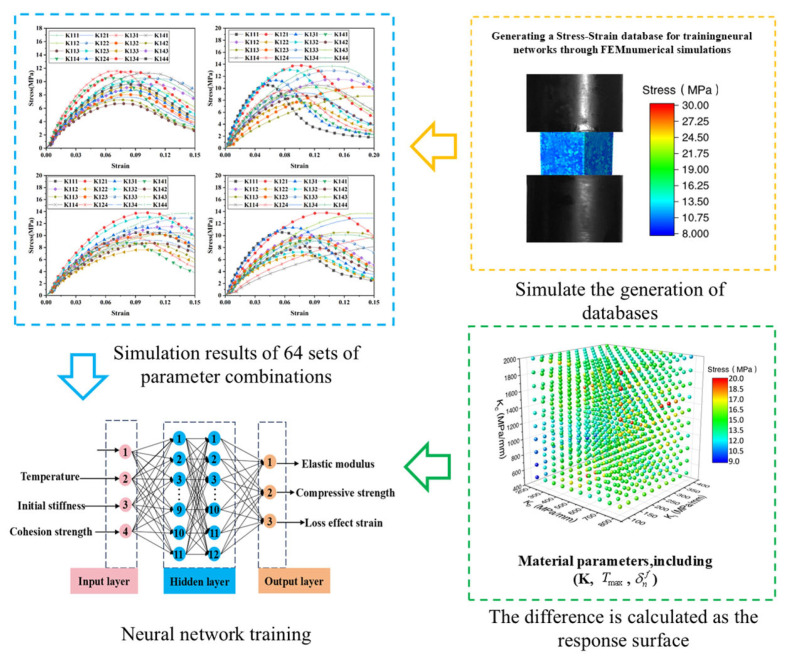
Schematic representation of the neural network training process using results from finite element simulations.

The bilinear cohesive zone model requires specification of three parameters for each interface: bond strength, critical displacement, and initial stiffness. Accordingly, a total of 12 parameters must be identified for the four considered interfaces. Accurate determination of these cohesive parameters necessitates an adaptive inverse algorithm characterized by rapid convergence and high precision. An artificial neural network (ANN) based on the back-propagation learning algorithm provides an efficient and accurate recursive solution. Figure 7 illustrates the training process of the ANN, which establishes a precise mapping between the stress–strain response and the cohesive parameters.

The ANN architecture comprises an input layer, two hidden layers, and an output layer. The input consists of dynamic compressive stress–strain curves extracted from the simulation database, while the output corresponds to the associated sets of cohesive parameters for each interface. Sigmoid functions serve as the activation functions for the hidden layers, and a Purelin linear function is employed in the output layer. The number of nodes in the hidden layers significantly affects network performance: insufficient nodes lead to large errors, whereas excessive nodes increase computational time. To balance accuracy and efficiency, each hidden layer was configured with 50 neurons. The network error (e) is calculated as follows:

(8) $$e=\frac{1}{2}\sum_{k=1}^{n}\left(O_{k}-T_{k}\right)$$

where O_k_ represents the calculated value, and T_k_ is the desired network output.

The ANN exhibits strong adaptability to complex nonlinear relationships. In this study, mean square error (MSE), R^2^, and root mean square error (RMSE) are selected as the performance evaluation metrics for the model. They are defined as follows:

(1) Mean square error

(9) $$MSE=\frac{1}{n}\sum_{i=1}^{n}\left(y_{i}-{\tilde{y}}_{i}\right)$$

where $y_{i}$ and ${\tilde{y}}_{i}$ represent the actual and predicted values of the ith sample, respectively, and *n* is the number of specimens.

(2) Coefficient of determination (R^2^)

R^2^ is the ratio of the sum of squares of the regression to the sum of squares of the total deviation in linear regression:

(10) $$R^{2}=1-\frac{\sum_{i=1}^{n}\left(y_{i}-{\tilde{y}}_{i}\right)}{\sum_{i=1}^{n}\left(y_{i}-{\bar{y}}_{i}\right)}$$

where ${\bar{y}}_{i}$ represents the average of the actual values, and its value equals the square of the correlation coefficient.

(3) Root mean square error

(11) $$RMSE=\sqrt{\frac{\sum_{i=1}^{n}{\left(y_{i}-{\tilde{y}}_{i}\right)}^{2}}{n}}$$

RMSE is a common metric used to measure the difference between values.

The training process utilized MATLAB2020’s neural network toolbox. Initially, the ANN was trained using sample data from a simulation database. Once training was complete, the inversion model was applied to estimate the cohesive parameters.

The procedure is outlined below:

Step 1: Define material property parameters for the training samples.

Step 2: Perform three-dimensional fine-grained compression simulations in ABAQUS to generate training data for the ANN. Use the resulting data to construct the simulation database.

Step 3: Input experimentally measured compressive stress–strain curves into the trained ANN model (Figure 7) to predict cohesive zone parameters.

Step 4: Estimate bond strength and critical displacement values using the MRS method, (The MRS method is an intelligent inversion strategy that combines response surface models with global optimization algorithms, specifically designed for identifying material constitutive parameters, such as interface strength and fracture energy of cohesive models). Its core idea is to iteratively construct high-precision surrogate models, which reduces computational costs while ensuring the robustness and global convergence of parameter identification) [41,42]. Initial stiffness values are determined through the inverse ANN model. Assign the optimized parameters to the propellant material model and perform dynamic uniaxial compression simulations in ABAQUS. Table 2 presents the inverted cohesive zone parameters for the four interfaces across different temperatures. Validation using SHPB experimental results confirms the reliability of the finite element simulations.

### 3.4. Experiment-Based Validation

**Figure 8:**
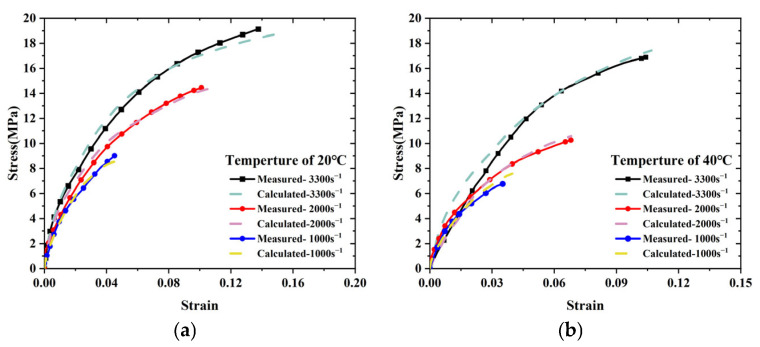

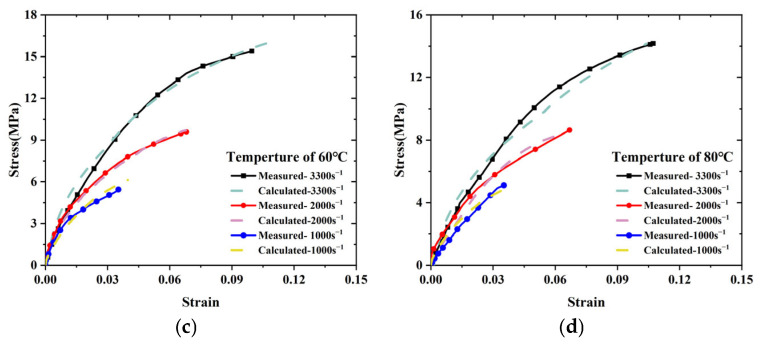
Comparison of experimental and simulated stress–strain curves at various temperatures and strain rates: (**a**) 20 °C. (**b**) 40 °C. (**c**) 60 °C. (**d**) 80 °C.

Figure 8 compares the computed and experimentally measured stress–strain curves for the solid propellant. The simulated results show strong qualitative agreement with experimental data, particularly in the elastic deformation region. The maximum deviation is limited to 7.3%, With reference to the error standards in *Standards for Verification and Validation in Computational Solid Mechanics* and *Guide to the Expression of Uncertainty in Measurement*, experimental results and numerical simulations with an error within 10% can be regarded as meeting the requirements of international standardization, indicating that the model accurately captures the material response and the identified parameters are valid.

**Figure 9:**
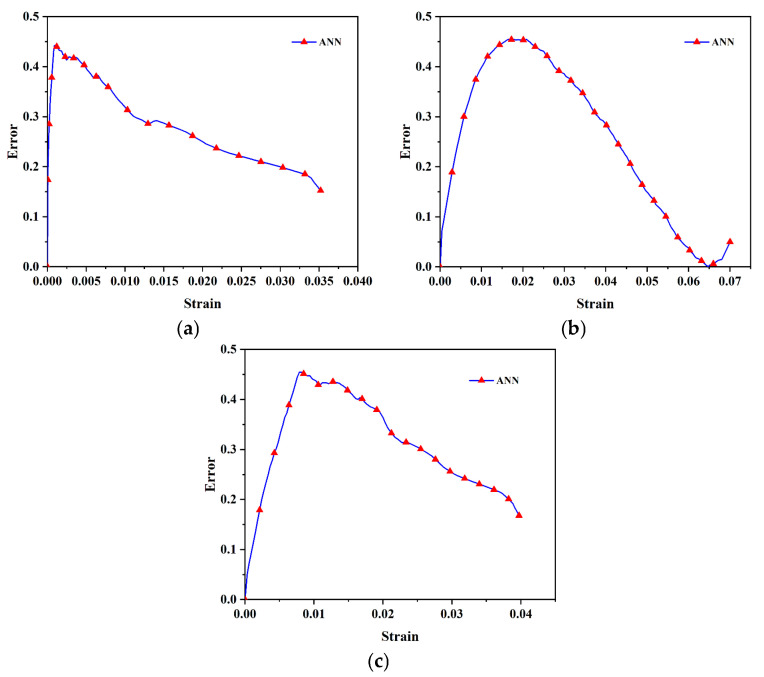
Prediction error of ANN model of training set at different loading rates at 60 °C: (**a**) 1000 s^−1^. (**b**) 2000 s^−1^. (**c**) 3300 s^−1^.

Regarding the training of the artificial neural network (ANN), our ANN model was trained and optimized using a total of 1728 synthetic data samples, with 70% allocated to the training set and 30% to the validation set. Based on the results of our previous material testing experiments, the ranges for relevant cohesive parameters were determined as follows: interface initial stiffness K_n_ ∈ [15, 150] MPa/mm, maximum traction S_n_ ∈ [5, 10] MPa, and initial damage displacement δ_n_ ∈ [0.0005, 0.005] mm. In this study, the ANN model was utilized to accurately predict the stress–strain behavior of the propellant under different temperatures and strain rates. To ensure the model’s reliability, a two-step validation process was implemented. First, the synthetic data (used for training) were split into a 70% training set and a 30% validation set to guide model optimization. Second, independent experimental data were randomly divided into a 70% training subset and a 30% testing subset for further validation; the trained ANN model was then applied to predict results for the testing subset. We conducted an error analysis on the cohesion parameters inverted by the neural network, as shown in Table 3 and Table 4. These tables present the error statistics of the interfacial cohesion parameters at 40 °C with a strain rate of 2000 s^−1^, including the mean squared error (MSE), root mean squared error (RMSE), and coefficient of determination (R^2^) for both the training set (synthetic data) and the testing set (experimental data). From these statistics, it can be calculated that the overfitting coefficient of each parameter is less than 1.2, which indicates that the training result is satisfactory. In addition, we introduced 5% Gaussian white noise into the ANN model and calculated the deviation between the parameters predicted by the ANN and the real parameters under the noisy data. The results show that the relative error is approximately 6.8% under 5% noise, indicating that the model is insensitive to noise and has strong robustness. To further demonstrate the model’s accuracy, we analyzed the prediction errors of the propellant at 60 °C under four different strain rates (Figure 9). The prediction errors decreased as the loading process progressed, highlighting the high precision of the ANN model in predicting the micromechanical properties of HTPB propellants.

## 4. Analysis and Discussion

### 4.1. Thermal Damage Behavior of Propellants at Different Temperatures

**Figure 10:**
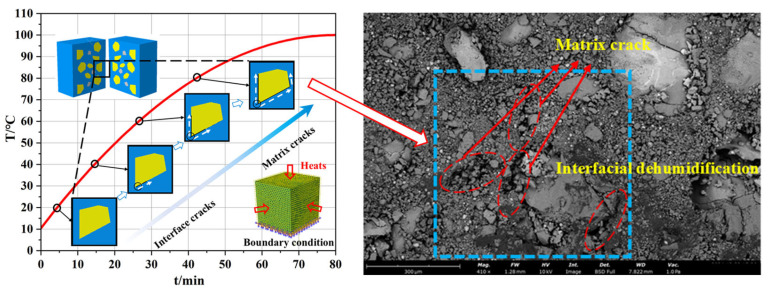
Thermal damage simulation results of the solid propellant at different temperatures.

A temperature–displacement coupling analysis was performed to establish a three-dimensional detailed model of the solid propellant, as depicted in Figure 10, this figure presents a comparison between the numerical simulation results of crack damage in solid propellants under different ambient temperatures and the SEM (Scanning Electron Microscope) images. The bottom surface was fully constrained, while thermal loads were applied to the remaining six surfaces. Based on experimental observations, to enable the propellant to stably heat up to different temperatures, we set a relatively slow heating rate. This ensures that the propellant can reach the various target temperatures for the study within 45 min. Meanwhile, we also set the same heating environment in the numerical simulation to guarantee the accuracy of the simulation. With increasing temperature, interfacial debonding emerged at the interface between the oxidizer particles and the binder. This process initiated the formation and gradual propagation of interfacial cracks. As illustrated in Figure 10, interfacial debonding was the initial damage mechanism. Subsequently, cracks nucleated at the tips of AP particles, evolved into matrix microcracks, and further propagated through the binder matrix.

### 4.2. Damage Behavior of Propellant at Different Strain Rates

**Figure 11:**
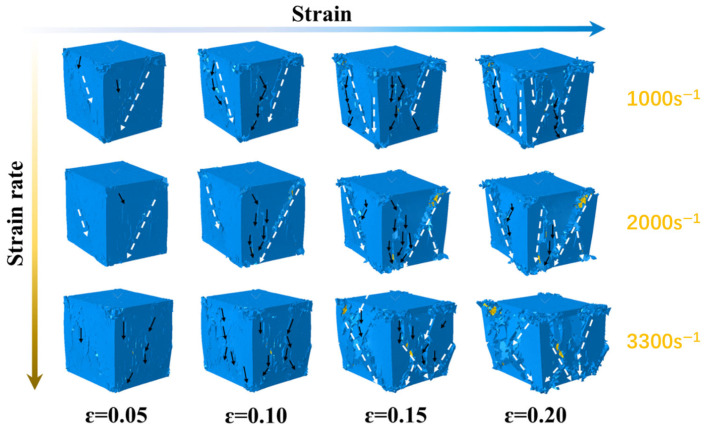
Deformation cloud diagrams of the solid propellant’s 3D fine-scale model under different 3 strain rates. Black dashed arrows indicate microcrack evolution trends; white arrows show the formation of macroscopic cracks.

**Figure 12:**
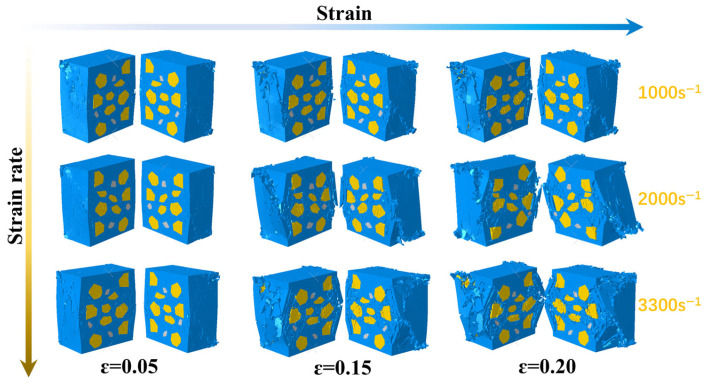
Cross-sectional damage profiles of the fine-scale model under varying strain rates.

The damage behavior of the solid propellant’s three-dimensional microscopic model under varying strain rates at a constant temperature was investigated. Figure 11 presents deformation cloud diagrams of the model at 20 °C for strain rates of 1000 s^−1^, 2000 s^−1^, and 3300 s^−1^. Black dashed arrows indicate the microcrack evolution trends, while white arrows highlight the formation of macroscopic main cracks caused by the coalescence of microcracks. Figure 12 displays the cross-sectional profile of Figure 10 at a position of 0.42 mm along the *x*-axis. This view clearly illustrates crack initiation and propagation within the oxidizer particles and binder matrix, revealing the complete evolutionary history of damage under compressive loading.

The damage evolution can be categorized into three distinct stages based on the deformation cloud and cross-sectional profile. In the first stage, elastic deformation occurs within the structure. The binder accommodates most of the compressive deformation due to its relatively low elastic modulus. The crystals exhibit minimal deformation. The second stage is marked by a nonlinear structural response. Strain concentrations develop in the binder due to lateral expansion, while the crystals remain largely undeformed. Once the surface stress of the binder exceeds its interfacial debonding strength, microcracks initiate. Strain energy localizes at the microcrack tips, leading to the development of shear slip zones. In polymeric binders, inter-chain shear slip contributes to plastic deformation and strain softening, resulting in reduced structural stiffness. As the binder’s plastic deformation capacity is exhausted, microcracks propagate, further compromising stiffness.

The third stage represents the post-peak damage evolution, during which microcracks begin to destabilize and propagate. Due to the high deformability of the binder, the cracks remain discontinuous and dispersed within the shear zone, rather than forming a dominant crack path. Significant crack tip blunting and pronounced strain localization are observed, consistent with previously reported findings [43].

**Figure 13:**
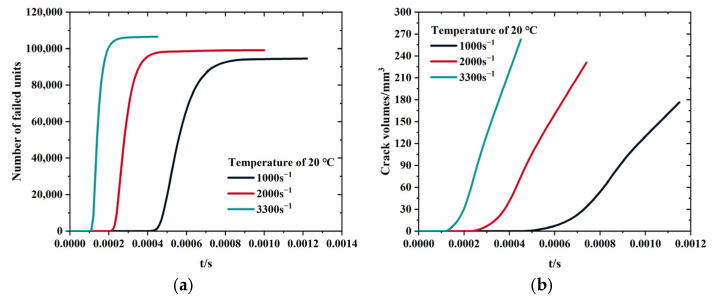
Crack-related parameters at different strain rates: (**a**) number of failed unit. (**b**) crack volume.

Crack propagation functions as a primary mechanism for strain energy release. Accordingly, variations in crack density and propagation rates reflect changes in the energy dissipation process. To quantify damage characteristics under different strain rates in the three-dimensional microscopic model of the solid propellant, the evolution of failed units and maximum crack width was recorded, as illustrated in Figure 13.

**Figure 14:**
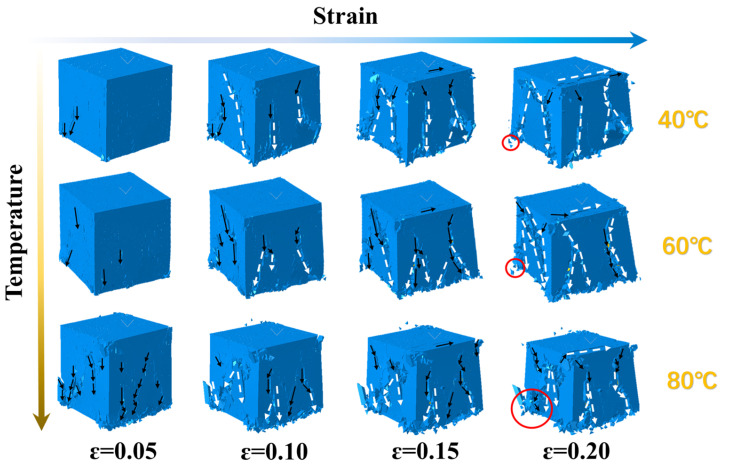
Damage cloud of the 3D fine-scale model of the solid propellant at different temperatures. The red circles denote regions of matrix damage.

Figure 14 illustrates a steady increase in crack volume and average crack propagation rate, indicating that the additional energy introduced by higher strain rates is predominantly dissipated through increased damage and faster crack propagation. Although both the number of failed units and propagation rates rise with strain rate, the overall increments remain moderate. As the strain rate increases, the propagation rate becomes more significant, while the number of failed units increases substantially. Specifically, under a compression strain rate of 3300 s^−1^, the crack propagation rate increases by 2.36 times, the number of failed units rises by 11.24%, and the crack volume expands by approximately 53.7%, relative to the values observed at 1000 s^−1^.

### 4.3. Damage Behavior of Propellant at Different Temperatures

**Figure 15:**
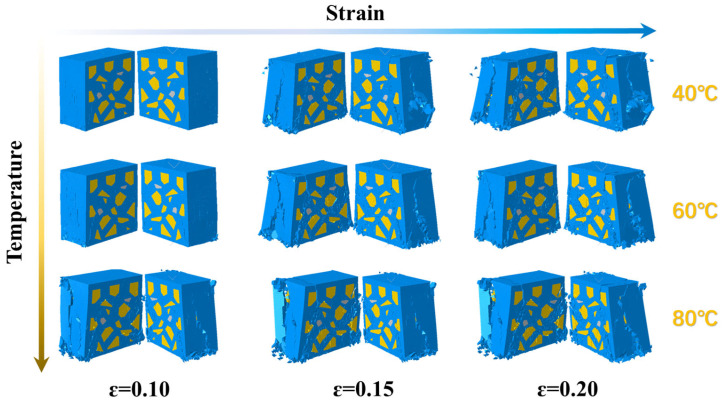
Detailed profiles of the modeled damage cloud for the solid propellant at different temperatures.

Figure 14 and Figure 15 present deformation cloud diagrams of the solid propellant at a constant strain rate of 1000 s^−1^ under ambient temperatures of 40 °C, 60 °C, and 80 °C. The deformation sequence under different thermal conditions proceeds through consistent stages: elastic deformation, interfacial displacement between particles and matrix, dewetting at the particle–matrix interface, and irreversible microcracking in the substrate. During the third stage, longitudinal elongation of the substrate increases significantly with temperature. Dewetting becomes more pronounced at the particle–matrix interface, resulting in enlarged void regions and the redistribution of mechanical loads to the matrix. This behavior indicates that, under identical strain rates, elevated temperatures induce enhanced dewetting, more complex microcrack evolution pathways in the matrix, and reduced material toughness. This softening effect promotes increased fragmentation. The observed trend is attributed to the elevated molecular mobility in the viscoelastic matrix at higher temperatures, which leads to shorter macroscopic stress relaxation times.

**Figure 16:**
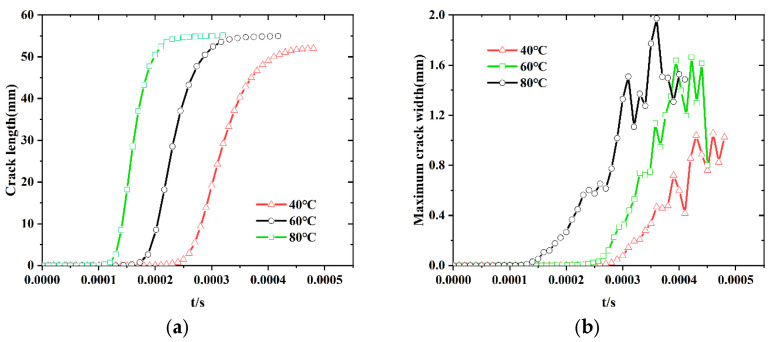

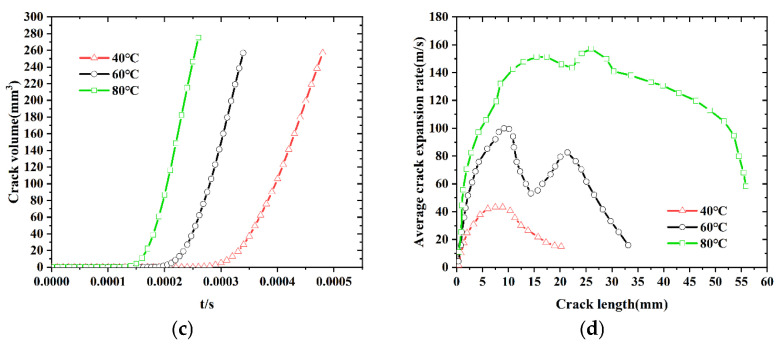
Crack extension parameters at different temperatures: (**a**) total crack length; (**b**) maximum crack width; (**c**) crack volume; (**d**) average crack tip expansion velocity.

Figure 16 presents the evolution of total crack length, crack volume, maximum crack width, and average crack tip expansion velocity as functions of impact time for the fine-scale solid propellant model at different temperatures. The term “crack tip” refers to cracks actively propagating at the current time step. The crack expansion rate was determined by differentiating the total crack length with respect to time, representing the cumulative propagation of all cracks in the model. The average crack expansion velocity, shown in Figure 16d, was obtained by dividing the total expansion rate by the number of active crack tips at each time step.

The data in Figure 16 indicate a gradual increase in both the number of crack tips and the average crack propagation velocity as temperature increases from 40 °C to 80 °C. This suggests that, at elevated temperatures, the strain energy introduced by dynamic loading is predominantly dissipated through increased crack tip formation and propagation rates. According to dynamic fracture theory, the Rayleigh wave velocity for the solid propellant is approximately 180 m/s. Despite increasing temperature, the peak crack propagation velocity remains below 200 m/s with limited fluctuation, indicating that propagation is constrained by the Rayleigh velocity. Therefore, the formation of additional crack tips, rather than an increase in individual crack speed, serves as the principal mechanism for strain energy dissipation at higher temperatures. Although the total crack length does not change significantly, the increase in crack tip density contributes to sustained fracture energy accumulation, thereby enhancing the system’s capacity for energy dissipation.

**Figure 17:**
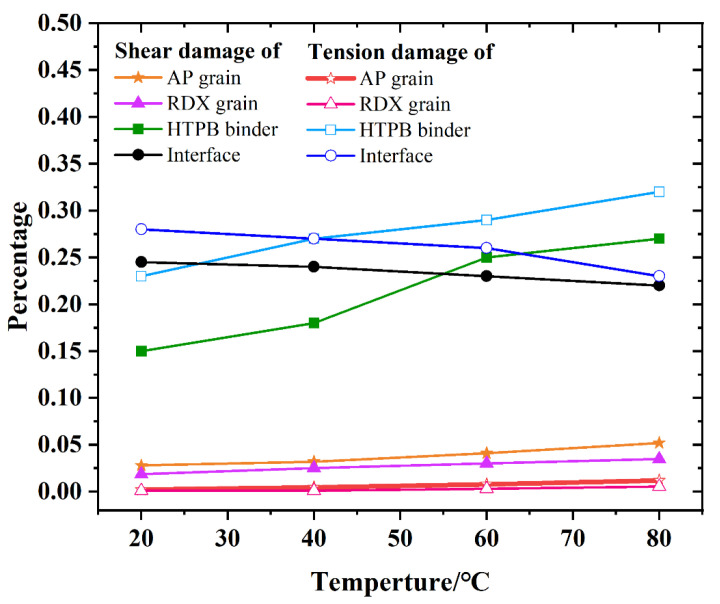
Distribution and proportion of failed cohesive units across four material components.

Interfacial debonding is widely recognized as the primary failure mode in solid propellants, with macroscopic strength strongly influenced by interfacial adhesion. Damage initiation predominantly occurs at the interface, while crack growth is largely driven by matrix deformation and fracture processes. The spatial distribution of failed units and the ratio of tensile to shear-induced damage in each component are presented in Figure 17. Across all temperature conditions, the matrix and interface exhibit a substantially higher concentration of damaged units than the particle regions. Specifically, the number of damaged units in the matrix increases with temperature, whereas the corresponding values for the interface and particles decrease. These observations indicate a temperature-driven transition in damage localization from the crystalline regions to the matrix and interface. This shift in failure mode is attributed to increased interfacial bond strength at elevated temperatures, which promotes greater matrix tearing and deformation between crystalline domains. As a result, a larger proportion of strain energy is dissipated through matrix fracture and viscoelastic deformation, thereby reducing stress concentration within the crystals and minimizing crystal rupture. However, as the matrix softens at higher temperatures, crystal regions within shear-dominant zones are subjected to enhanced compressive forces and collisions due to matrix slip. Under such conditions, compressive shear becomes the dominant damage mechanism in the crystals. These findings align with previous observations reported by Wiegand and Pinto [44]. Furthermore, the increased proportion of tensile failure at the interface suggests that tensile deformation predominates in this region. Given that mode I fracture toughness is the lowest among fracture modes, cracks are most likely to initiate at the interface. With increasing temperature, a decline in the number of fractured crystals is observed. Therefore, the post-peak damage behavior of the material exhibits more ductile characteristics, as reflected by a reduced slope in the post-peak segment of the stress–strain curve.

## 5. Conclusions

This study presents a novel methodology that integrates artificial intelligence-based inverse identification algorithms with three-dimensional cohesive zone modeling to investigate the damage mechanisms in solid propellants under coupled thermal and mechanical loading conditions. The analysis reveals that energetic solid propellants exhibit pronounced sensitivity to both strain rate and temperature. The primary conclusions are as follows:

(a) Increasing temperature significantly increases the number of crack tips, and the fracture energy growth rate exceeds the crack propagation rate limit. This enables efficient dissipation of strain energy at elevated temperatures.

(b) At higher temperatures, dewetting and fragmentation become more severe under equivalent strain levels. These effects are accompanied by more complex microcrack networks in the matrix, indicating a marked reduction in material toughness (softening behavior).

(c) The mode of crack propagation is strongly temperature-dependent. Lower temperatures are associated with higher crack velocities and longer total crack lengths, while higher temperatures promote an increase in crack tip density. This shift enhances peak fracture energy and its growth rate, facilitating effective energy dissipation through this mechanism.

## Figures and Tables

**Table 1:** Prony series fitting parameters for the HTPB relaxation modulus master curve.

*i*	Log (*E*(*t*))	Log ($\tau_{i}$)	*i*	Log (*E*(*t*))	Log ($\tau_{i}$)
1	2.213	−9	10	1.112	0
2	2.072	−8	11	1.063	1
3	1.896	−7	12	1.038	2
4	1.731	−6	13	0.982	3
5	1.588	−5	14	0.931	4
6	1.541	−4	15	0.858	5
7	1.452	−3	16	0.816	6
8	1.336	−2	17	0.724	7
9	1.235	−1	18	0.619	8

**Table 2:** Cohesive zone model parameters for particles, matrix, and interfaces.

Type	Property	20 °C	40 °C	60 °C	80 °C
AP Particle	Initial stiffness *K*_n_ (MPa/mm)	647	580	520	460
	Maximum traction *S_n_* (MPa)	32.4	30.2	28.6	26.5
	Critical separations *δ_n_*, *δ_t_* (mm)	0.008	0.007	0.006	0.005
RDX Particle	Initial stiffness *K*_n_ (MPa/mm)	820	780	740	650
	Maximum traction *T_n_* (MPa)	52.4	48.2	46.4	44.3
	Critical separations *δ_n_*, *δ_t_* (mm)	0.01	0.009	0.008	0.007
HTPB Binder	Initial stiffness *K*_n_ (MPa/mm)	368	282	221	165
	Maximum traction *S_n_* (MPa)	12.7	10.6	8.6	6.4
	Critical separations *δ_n_*, *δ_t_* (mm)	0.005	0.004	0.003	0.002
Interface	Initial stiffness *K*_n_ (MPa/mm)	134	88	42	21
	Maximum traction *S_n_* (MPa)	9.8	8.1	7.2	5.7
	Critical separations *δ_n_*, *δ_t_* (mm)	0.003	0.002	0.0015	0.001

**Table 3:** Performance summary of Ann model of the training set.

Evaluation Index	MSE	RMSE	R^2^
Initial stiffness K_n_ (MPa/mm)	0.0465	0.232	0.976
Maximum traction S_n_ (MPa)	0.142	0.383	0.955
Critical separations δ_n_, δ_t_ (mm)	1.32 × 10^−8^	1.1 × 10^−4^	0.963

**Table 4:** Performance summary of Ann model of the testing set.

Evaluation Index	MSE	RMSE	R^2^
Initial stiffness K_n_ (MPa/mm)	0.0476	0.245	0.948
Maximum traction S_n_ (MPa)	0.145	0.396	0.941
Critical separations δ_n_, δ_t_ (mm)	1.57 × 10^−8^	1.3 × 10^−4^	0.951

## Data Availability

The data presented in this study are available on request from the corresponding author.

## References

[1] Zou Z., Qiang H., Zhang F., Wang X., Li Y. (2025). Research on mechanical behavior of particle/matrix interface in composite solid propellant. Eur. J. Mech.-A/Solids.

[2] Xing R., Wang L., Zhang F., Hou C. (2022). Mechanical behavior and constitutive model of NEPE solid propellant in finite deformation. Mech. Mater..

[3] Gouhier F., Diani J., Vandenbroucke A. (2024). A finite strain viscoelastic model with damage and tension–compression asymmetry considerations for solid propellants. Mech. Mater..

[4] Kokash Y., Regueiro R., Miller N., Zhang Y. (2024). A non-isothermal breakage-damage model for plastic-bonded granular materials incorporating temperature, pressure, and rate dependencies. Int. J. Solids Struct..

[5] Wang G., Wu Y., Yang K., Xia Q., Huang F. (2024). Optimization of mechanical and safety properties by designing interface characteristics within energetic composites. Def. Technol..

[6] Lu D., Zhang B., Liu L., Zhang H., Cao L., Zhou Y. (2025). Three-dimensional cohesive finite element simulations coupled with machine learning to predict mechanical properties of polymer-bonded explosives. Compos. Sci. Technol..

[7] Chaturvedi S., Dave P.N. (2012). Nano-metal oxide: Potential catalyst on thermal decomposition of ammonium perchlorate. J. Exp. Nanosci..

[8] Arora H., Tarleton E., Li-Mayer J., Charalambides M., Lewis D. (2015). Modelling the damage and deformation process in a plastic bonded explosive microstructure under tension using the finite element method. Comput. Mater. Sci..

[9] Zhang X., Luo G., Zhou H., Wei Q., Yang X., Zhu Y., Cao P., Shen Q. (2024). Damage behavior of high particle volume fraction composites with initial damage by finite element meso-modeling. Compos. Struct..

[10] Yang D.O., Qin C., Qin X., Xie Q., Nian X. (2024). The thermal response of the ignition and combustion of high-energy material projectiles under the influence of heat. Case Stud. Therm. Eng..

[11] Guo Z., Xu J., Chen X., Wang T., Liu J., Zhang H., Chen Y., Song Q. (2024). Effects of dynamic loading and temperature on NEPE propellant: Damage and ignition analysis. Mech. Time-Depend. Mater..

[12] Geng T., Qiang H., Wang Z., Wang X., Zhu Z., Qiao D. (2023). Macroscopic and mesoscopic properties of HTPB propellant under low temperature dynamic biaxial compression loading. Polym. Test..

[13] Keyhani A., Kim S., Horie Y., Zhou M. (2019). Energy dissipation in polymer-bonded explosives with various levels of constituent plasticity and internal friction. Comput. Mater. Sci..

[14] Deng X., Huang Y., Zhao J. (2024). Numerical investigation of damage and ignition behaviors of PBX under punch loading. Eng. Fract. Mech..

[15] Xia Q.-Z., Wu Y.-Q., Huang F.-L. (2023). Effect of interface behaviour on damage and instability of PBX under combined tension–shear loading. Def. Technol..

[16] Rae P., Goldrein H., Palmer S., Field J., Lewis A. (2002). Quasi–static studies of the deformation and failure of β–HMX based polymer bonded explosives. Proc. R. Soc. London. Ser. A Math. Phys. Eng. Sci..

[17] Lei M., Wang J., Cheng J., Xiao J., Wen L., Lu H., Hou X. (2020). A constitutive model of the solid propellants considering the interface strength and dewetting. Compos. Sci. Technol..

[18] Oberth A., Bruenner R. (1965). Tear phenomena around solid inclusions in castable elastomers. Trans. Soc. Rheol..

[19] Hou Y., Xu J., Zhou C., Chen X. (2021). Microstructural simulations of debonding, nucleation, and crack propagation in an HMX-MDB propellant. Mater. Des..

[20] Guo Y., Liu R., Chen P., Zhou B., Hu G., Han C., Lv K., Zhu S. (2022). Mechanical behavior of PBX with different HMX crystal size during die pressing: Experimental study and DEM simulation. Compos. Sci. Technol..

[21] Liu C., Shi Y., Liang X. (2014). DEM study on hot spots formation of heterogeneous explosives under shock loading. Chin. J. Comput. Phys..

[22] Ahmadi M., Sadighi M., Hosseini-Toudeshky H. (2022). Microstructure-based deformation and fracture modeling of particulate reinforced composites with ordinary state-based peridynamic theory. Compos. Struct..

[23] McDonald B.A., Rice J.R., Kirkham M.W. (2014). Humidity induced burning rate degradation of an iron oxide catalyzed ammonium perchlorate/HTPB composite propellant. Combust. Flame.

[24] Di Benedetto G.L., van Ramshorst M.C., Duvalois W., Hooijmeijer P.A., van der Heijden A. (2017). In-situ tensile testing of propellants in SEM. Propellants Explos. Pyrotech..

[25] Hu R., Prakash C., Tomar V., Harr M., Gunduz I.E., Oskay C. (2017). Experimentally-validated mesoscale modeling of the coupled mechanical–thermal response of AP–HTPB energetic material under dynamic loading. Int. J. Fract..

[26] de Francqueville F., Gilormini P., Diani J., Vandenbroucke A. (2020). Comparison of the finite strain macroscopic behavior and local damage of a soft matrix highly reinforced by spherical or polyhedral particles. Eur. J. Mech.-A/Solids.

[27] Liu Y., Qian W., Wang L., Xue Y., Hou C., Wu S. (2023). In situ X-ray tomography study on internal damage evolution of solid propellant for carrier rockets. Mater. Sci. Eng. A.

[28] Dai K., Lu B., Chen P., Chen J. (2019). Modelling microstructural deformation and the failure process of plastic bonded explosives using the cohesive zone model. Materials.

[29] Hu Z., Zhang K., Liu Q., Wang C. (2024). NEPE Propellant Mesoscopic Modeling and Damage Mechanism Study Based on Inversion Algorithm. Materials.

[30] Zhang X., Chang X., Lai J., Hu K. (2013). Comparative research of tensile and compressive mechanical properties of HTPB propellant at low temperature. J. Solid Rocket Technol..

[31] Xu Y., Zhao S., Jin G., Wang X., Liang L. (2017). Ductile fracture of solder-Cu interface and inverse identification of its interfacial model parameters. Mech. Mater..

[32] Xiao Y., Sun Y., Yang Z., Guo L. (2017). Study of the dynamic mechanical behavior of PBX by Eshelby theory. Acta Mech..

[33] Xu X.-P., Needleman A. (1994). Numerical simulations of fast crack growth in brittle solids. J. Mech. Phys. Solids.

[34] Gu X., Liu X., Dong C., Zhang G., Zhang L., Zhang F. (2023). The Mesoscopic Numerical Simulation of GAP/CL20/AP Composite Solid Propellant Based on MPM and FEM. Appl. Sci..

[35] Li M.-M., Li F.-S., Shen R.-Q. (2011). Molecular dynamics study of RDX/AMMO propellant. Chin. J. Chem. Phys..

[36] Xiao Y.C., Sun Y., Li X., Zhang Q., Liu S., Yang H. (2016). Dynamic mechanical behavior of PBX. Propellants Explos. Pyrotech..

[37] Gergesova M., Zupančič B., Saprunov I., Emri I. (2011). The closed form tTP shifting (CFS) algorithm. J. Rheol..

[38] Ho S.-Y. (2002). High strain-rate constitutive models for solid rocket propellants. J. Propuls. Power.

[39] Xu J., Chen X., Wang H., Zheng J., Zhou C. (2014). Thermo-damage-viscoelastic constitutive model of HTPB composite propellant. Int. J. Solids Struct..

[40] Duncan E., Margetson J. (1998). A nonlinear viscoelastic theory for solid rocket propellants based on a cumulative damage approach. Propellants Explos. Pyrotech..

[41] Prakash C., Gunduz I.E., Oskay C., Tomar V. (2018). Effect of interface chemistry and strain rate on particle-matrix delamination in an energetic material. Eng. Fract. Mech..

[42] Olokun A.M., Prakash C., Emre Gunduz I., Tomar V. (2020). The role of microstructure in the impact induced temperature rise in hydroxyl terminated polybutadiene (HTPB)–cyclotetramethylene-tetranitramine (HMX) energetic materials using the cohesive finite element method. J. Appl. Phys..

[43] Ambos A., Willot F., Jeulin D., Trumel H. (2015). Numerical modeling of the thermal expansion of an energetic material. Int. J. Solids Struct..

[44] Wiegand D.A., Pinto J.J. (1995). The composition of polymer composite fracture surfaces as studied by XPS. MRS Online Proc. Libr. (OPL).

